# Interpretable prediction and generation of ASC-speck aptamers using multiscale deep biological learning models

**DOI:** 10.1093/bioadv/vbag168

**Published:** 2026-06-13

**Authors:** Mengting Niu, Quan Zou, Lei Xu

**Affiliations:** Institute of Fundamental and Frontier Sciences, University of Electronic Science and Technology of China, Chengdu 610054, China; Yangtze Delta Region Institute (Quzhou), University of Electronic Science and Technology of China, Quzhou 324000, China; Institute of Fundamental and Frontier Sciences, University of Electronic Science and Technology of China, Chengdu 610054, China; Yangtze Delta Region Institute (Quzhou), University of Electronic Science and Technology of China, Quzhou 324000, China; School of Electronic and Communication Engineering, Shenzhen Polytechnic University, Shenzhen 518055, China

## Abstract

**Motivation:**

Aptamers are functional nucleic acids that can bind to corresponding ligands and effectively replace monoclonal antibodies. However, some target proteins may lack binding candidate DNA sequence, necessitating methods to generate new aptamer libraries for out-of-donor detection. Therefore, we propose an adaptive prediction and design method, ASC2BF for DNA aptamer generation.

**Results:**

Based on the multiscale residual network, ASC2BF not only predicts the aptamers simultaneously based on the characteristics of DNA–protein aptamers, but also uses the bacterial foraging optimization algorithm (BFOA) to generate new DNA aptamer sequences based on biophysical constraints, with initial seeds screened by the neural network predictor. As a case study, ASC2BF was applied to generate aptamer pools against apoptosis-associated speck-like proteins (ASC-speck). Furthermore, we demonstrate the ability of deep learning to capture sequential and functional semantic information. And through interpretability analysis, our results demonstrate what the model learns, helping us build a map from discovering important information to analyzing its biological function.

**Availability and implementation:**

The code and data sets are obtainable at https://github.com/nmt315320/aptamer.git.

## 1 Introduction

Aptamers are RNA/DNA oligonucleotide molecules that bind specifically to targeted complementary molecules. Compared to antibodies, aptamers require fewer target constraints, enabling their application in various treatments and diagnostic procedures for blood disorders such as hematoma and hemophilia (Cucchiarini *et al.* 2025). Aptamers offer several advantages over antibodies, including higher specificity, easier production, applicability to diverse target types, better stability, and favorable storage properties (Jayasena 1999, Groff *et al.* 2015, Gupta *et al.* 2025). These characteristics make aptamers promising tools for diagnosis and treatment (Zhou and Rossi 2017). Aptamers have been widely used and studied in many fields of biomedicine (Ștefan *et al.* 2021, Stangherlin *et al.* 2025), such as biomarker detection (Chang *et al.* 2013), imaging (Englert *et al.* 2023), and targeted therapy (Vandghanooni *et al.* 2018). Several aptamers have been developed over the past 20 years (Adachi and Nakamura 2019, Wang *et al.* 2024). For example, pegaptanib was the first FDA approved drug for the treatment of vascular diseases of the eye (Bege *et al.* 2025). AS1411 was the first aptamer to be tested in human clinical trials (Soundararajan *et al.* 2008). The success of pegaptanib provides ample evidence for its potential use as a novel therapy.

In 1990, Tuerk first developed aptamers in the Exponential Enriched ligand Phylogenetic Evolution (SELEX) study (Tuerk and Gold 1990). The SELEX procedure is a screening technique for nucleic acid aptamers that used purified target molecules, starting from an initial library, through a cycle of target binding, selection, and amplification (Kohlberger and Gadermaier 2022). SELEX may require 10–20 rounds to enrich and identify aptamers that bind to the target, resulting in a complex and time-consuming process of screening from nucleic acid molecular libraries (Preeti *et al.* 2024). To shorten the time required for appropriate screening and improve the success rate of screening, many improvements have been made to the traditional SELEX technique. For example, Krylov’s group developed a nonequilibrium capillary electrophoresis technique for balancing mixtures that can shorten the fitter period for screening targets compared to the traditional SELEX technique (Berezovski *et al.* 2005). Hung *et al.* developed a microfluidic system that can use automated tissue SELEX and phage display techniques for ovarian cancer tissue sections (Hung *et al.* 2018). Despite these advances, most published aptamers are still identified through manual observation of oligonucleotide binding enrichment, a labor-intensive process.

With advances in statistics and computational science, researchers are increasingly interested in applying statistical methods and artificial intelligence to make adaptable predictions and to build computing tools to further the research on the aptamer–target interaction. There are two steps in the calculation process of the development of an aptamer: aptamer–target interaction prediction and aptamer pool design. For interaction prediction, the development method is only used as a classifier to evaluate the appropriate aptamer–target interaction. There are two main methods: interaction-based predictions (Chen *et al.* 2021) and structural predictions (Emami *et al.* 2020). By considering the physical chemistry, energy status, and structural characteristics of an aptamer, an in-depth understanding of the mechanism of interaction between aptamer and its goals can be obtained (Yang *et al.* 2019, Heredia *et al.* 2021, Shin *et al.* 2023). Emami and Ferdousi (2021) proposed a deep neural network that integrates the features of ligands and target proteins to predict the binding interactions between ligands and proteins. Junhua Gu integrated AdaBoost and random forest (RF) to predict the interactions between ligands and proteins–ligands as well as between proteins and ligands based on the key sequence features of proteins and ligands (Li *et al.* 2020). Qing Song proposed an integrated method to predict aptamer–protein interactions with hybrid features (Zhang *et al.* 2016). Chushak and Stone (2009) used the secondary structural prediction and calculation pair to screen the start sequence pool. Knight *et al.* (2009) used machine learning to simulate sequence adaptation by training RF models in aptamer and its corresponding affinity. The prediction model based on experimental results has successfully calculated the yeast UTR sequence with higher protein expression and an antibody sequence with high specificity. The machine learning model can help researchers distinguish potential DNA aptamers, to significantly reduce the number of sequences that need to be screened, and provide the basis for the next step based on SELEX data analysis (Ahmad *et al.* 2021). Yang *et al.* (2019) integrated the sequence features of aptamers and target proteins to predict aptamer–protein interaction pairs and proposed a new integrated method. Li *et al.* integrated the machine learning framework of adaboost and RF to develop a server for predicting aptamers and protein–aptamer and protein/aptamer. Zhang proposed an integrated approach to predict aptamer–protein interaction pairs with hybrid features. Neda Emami proposed a new deep neural network for predicting aptamer–protein interaction pairs by integrating the characteristics of ligands and target protein. Ruiz-Ciancio *et al.* (2024) proposed an aptamer clustering algorithm to select ranked or scored candidates for experimental validation by providing context to describe the aptamer group. Tanmoy Som proposed a new intuitionistic fuzzy rough feature selection method based on *k*-means, which enhances the prediction of the interaction between the ligand and its target protein by using the sequence features obtained from the ligand and its target protein (Jain *et al.* 2024). Yuanyuan Yu proposed a hybrid neural network model that combines convolutional neural networks and bidirectional long short-term memory. The model predicts the binding affinity and potential binding motifs of ligands by integrating sequence composition and structural features (Yang *et al.* 2025). These developments motivate the application of advanced deep learning techniques to improve predictive models by integrating data from multiple experimental platforms.

ASC-speck is a kind of connecting protein in the inflammatory body, that are formed from a macromolecular diode concentrated from ASC protein with a molecular weight of 22 kDa (Stutz *et al.* 2013). The ASC-speck generated in this way will cause a large amount of messenger substances to form between cells and “ask for help” from the immune system(Jenster *et al.* 2023). The formation of ASC-speck is essential for the activation of Caspase-1. Regulating the formation of ASC-speck is a new approach to treating and preventing disease-related to other diseases (Chaturvedi *et al.* 2022). Aptamers are typically nongenomic but biologically active single-stranded nucleic acid molecules, usually between 10 and 100 nucleotides in length. They can be designed to have a variety of molecular targets with high affinity and specificity.

**Figure 1:**
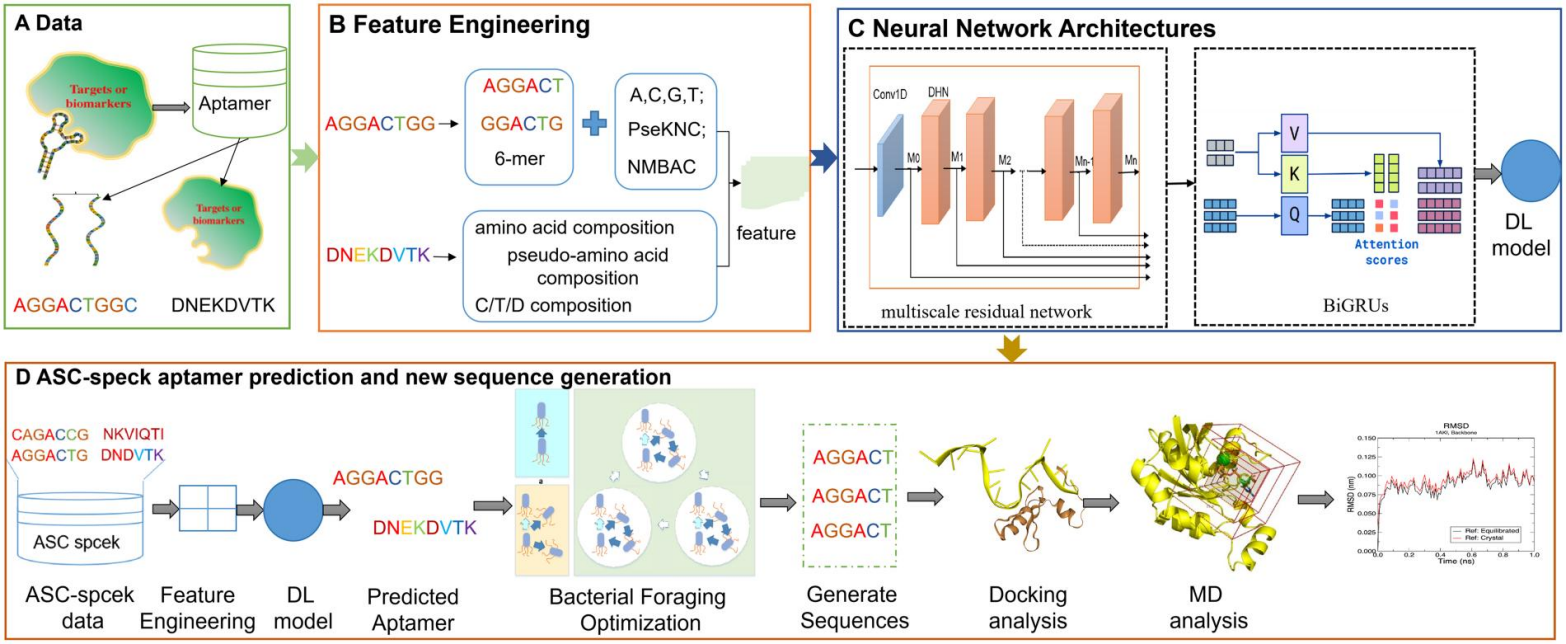
Overview of the proposed ASC2BF framework. (A) Data collection: target protein, biomarkers, and aptamer sequences. (B) Feature engineering: extraction of 6-mer, NMBAC, Pse-KNC, and other sequence features. (C) Neural network architectures: multiscale residual network (MRN), BiGRUs, and attention mechanism for deep learning-based classification. (D) ASC-speck aptamer prediction and new sequence generation: using the trained DL model to predict aptamer candidates, followed by bacterial foraging optimization algorithm (BFOA) to generate new sequences.

Inspired by intelligent learning algorithms (Wang *et al.* 2024), we downloaded genome sequences and treated sequence determinants at different scales as distinct biological vocabularies. Based on this idea, we propose ASC2BF, a multiscale residual neural network for identifying DNA aptamers and designing new ones. The overall framework is illustrated in Fig. 1. Specifically, we introduce a multiscale processing strategy to capture discriminative information across different scales and employ a multiscale residual network (MRN) to learn biological context semantics. Using the predicted aptamer–target protein interactions, we construct an initial seed library of aptamers, and then apply the bacterial foraging optimization algorithm (BFOA) to generate novel aptamer sequences. To address the issue of imbalanced training data, we adopt online hard example mining (OHEM). We validate the proposed method through a case study on ASC-speck proteins, and analyze the generated sequences using docking and molecular dynamics (MD) simulations. Experimental results demonstrate that ASC2BF effectively explores the aptamer design space and achieves promising performance.

## 2 Materials and methods

### 2.1 Data

The dataset S consists of two parts S1 and S2. The aptamers selected were DNA aptamers with unmodified bases and backbones. The DNA aptamer sequences were obtained from the database Aptagen (Muppirala *et al.* 2011) using Python. After cleaning and deleting the modified base and duplicate sequence, 238 aptamer sequences were obtained (this part was designated S1). In previous studies, Li *et al.* (2014) built datasets, including 328 aptamers, downloaded from the Aptamer Base database (Cruz-Toledo *et al.* 2012), based on PubMed through the search “SELEX” or “Aptamer”(this part was designated S2). The two datasets were combined, repeated sequences were deleted, and 565 DNA aptamers were finally obtained.

Then there was the acquisition of negative sample. The DNA data were obtained from a customized Python script from the nucleic acid database. Then a customized script was implemented for data processing (i.e. clearing and deleting the sequence of modified alkali base and copy). Finally, we obtained a dataset which contains 4885 sequences which can be obtained in https://github.com/nmt315320/aptamer.git. The dataset was divided at a ratio of 8:2 to divide the training set and test set.

It is noteworthy that the imbalance of the dataset will affect the predictive performance of the predicted model. There were two solutions for this problem. One method was to use the SMOTE (Chawla *et al.* 2002, Niu *et al.* 2021) algorithm to change the original imbalanced data. Another more valid method was to measure the loss of each class at the end of the network. Because directly generating the data may reduce the credibility of the original data set. Therefore, we should choose the second method to solve the problem of imbalance. We first assigned a class weight inversely proportional to the number of valid samples for each class. Subsequently, based on OHEM (Shrivastava *et al.* 2016), we designed the label model, which was realized by learning the weight of each task during training. We also analyzed the capability of the focus loss, which basically reduced the weight of the loss of the losses associated with samples with good classification.

### 2.2 Feature engineering

#### 2.2.1 Aptamer representation

In ASC2BF, we used word2vec (Church 2017) to extract the textual semantics of tagged DNA sequences. In this way, each sequence was segmented into words of fixed length. Previous studies have shown that 6-mer performs better than other lengths in appropriate target identification. So, we used 6-mer to process the input sequence and learn richer embedding information. For example, for the sequence R, divide it into the words w1, w2, …, w*n* by the length of 6-mer. R was expressed by the total probability formula. Then, the probability of the occurrence of the target word was estimated through the context of length *n*, and it was mapped into the space.

In addition to word2vec, other characteristics of each nucleotide sequence were also calculated. These features include: single nucleotide composition (SAC), pseudo nucleotide composition (Pse-KNC), and Moreau-Broto (NMBAC). SAC calculated the frequency of each nucleotide (A, C, G, T/U) in a nucleic acid sequence. Pse-KNC was calculated according to the physicochemical properties of the aptamer, so it can also indicate the physicochemical properties of the appropriate ligand and effectively represent the aptamer information. NMBAC was extracted from 11 smoothing properties (shift, slide, rise, tilt, roll, twist, superposition energy, superposition energy, superposition, entropy, free energy, hydrophilicity).

#### 2.2.2 Protein representation

After extensive experiments and screening, we determined the protein features that needed to be extracted in the study, including amino acid composition, pseudo-amino acid composition, group amino acid composition, C/T/D composition, physicochemical properties, and sequence coupling number. C/T/D is the distribution pattern of amino acids with specific structure or physicochemical properties, and its composition refers to the ratio of amino acids with specific properties to the total number of amino acids, and the physicochemical properties of seven amino acids were studied, which are hydrophobicity, secondary structure, solubility, normalized van der Waals volume, polarity, polarization, and charge. The sequence coupling number is the distance between two amino acids calculated from the physical and chemical distance matrix of amino acids. The distance matrix adopts Schneider–Wrede’s physical chemical matrix and Grantham’s chemical matrix.

### 2.3 Neural network architectures

The model was mainly composed of two parts: deep MRN and BiGRUs network with attention mechanism. The model can automatically extract local and global deep features from shallow features. The specific structure is shown in Fig. 1B–F. During the training process, the Adam optimizer was employed for parameter optimization. Each method and dataset was run for 300 epochs, and the epoch with the highest accuracy on the validation set was selected to determine the final test accuracy. To facilitate model tuning, the following hyperparameters were set: a dropout rate of 0.5, a weight decay coefficient of 5e−4, a consistency loss weight of 0.6, and hidden layer dimensions of {64, 32, 16}.

The deep MRN (Niu *et al.* 2022) was initially applied to image classification and achieved good results. MRN employs convolution kernels of different sizes (or dilated convolutions with different dilation rates) to process the input sequence in parallel, thereby capturing context information from local to global: small-scale convolution: captures nucleotide-dependent relationships over short distances; large-scale convolution: captures sequence interactions over long distances; multiscale feature fusion: concatenates or adds feature maps of different scales to form a comprehensive representation containing multi-granularity information.

Besides, ResNet can solve the degradation problem of the deep network by learning to fit the residual relative to the output of the previous layer. To avoid gradient disappearance and overfitting, a pre-activated ResNet with identity mapping was used in our model to skip connections and add after activation. Because the addition of “jump connection” makes the gradient flow back to any shallow layer through “1” to avoid the gradient dispersion caused by the convolution layer, the problem of network degradation is well solved. The ResNet enables the gradient to be directly passed back to the shallow layers, allowing the network to be stacked deeper and enabling it to learn more abstract semantic features.

The BiGRUs layer extracted the characteristics of the input data by connecting the forward GRU layer and the reverse GRU layer. Each BiGRU unit consisted of a forward GRU unit and a reverse GRU unit. Bidirectional GRUs can adaptively change state based on input to solve the problem of gradient disappearance in RNN.

The self-attention adaptively learnt important parts, expressed importance information by calculating weight, assigned more weight to key features, and ignored unimportant parts. In the ASC2BF model, the output matrix of BiGRU layer was input to the attention layer, and different features were given different weights, and attention mechanism was used to give greater weights to key features, so as to ensure that the model can be more accurate aptamer recognition.

To clarify the operational meaning of “biological context semantics” and how the MRN learns it, we provide the following details. The MRN in ASC2BF is designed to capture multiscale sequence patterns that are predictive of aptamer–target binding. It takes as input the feature vectors generated from the encoding module and outputs a high-dimensional semantic embedding. This embedding is then fed into the BiGRU-attention layers for classification.

### 2.4 Performance metrics

In this study, we evaluate the performance of ASC2BF using the following metrics: accuracy (ACC), Matthews correlation coefficient (MCC), sensitivity (SE), and specificity (SP). The formulas of these indicators are described as follows


(1) {SE=TPTP+FNSP=TNTN+FPACC=TN+TPTN+FP+TP+FNMCC=TP×TN-FP×FN(TP+FP)×(TP+FN)×(TN+FP)×(TN+FN)

Both ACC and MCC are used to measure the overall performance of ASC2BF. In addition, the overall predictive performance of the model is visually evaluated using the ROC (receiver operating characteristic) curve. AUC, the area under the ROC curve, is used to quantitatively measure the overall performance of ASC2BF.

### 2.5 Creation of ASC-speck-binding aptamer pool using BFOA

To predict the adaptation of the ASC-speck target protein, we obtained 150 ASC-speck protein sequences from UniProt. The combination of ASC-speck protein and DNA feature vectors was used as the input of ASC2BF predictive models, and the probability of a DNA sequence combined with ASC-speck target protein was higher. The higher the score, the greater the possibility of combining with the target protein. Therefore, the DNA sequence that can be combined with ASC-speck was identified. Then, based on the DNA-encoded algorithm, BFOA was used to optimize the design of DNA sequence coding (Ren and Yao 2018), set optimization goals, and generate new adapter sequences on the best adaptive seed ancestors according to the level of evaluation indicators. It is important to note that the BFOA fitness function (Eq. (8)) relies solely on biophysical constraints (Tm, GC%, Sim, Hm, Con, Hair) and does not incorporate any score from the MRN predictor. The MRN is used only for aptamer classification, not for guiding sequence generation.

### 2.6 BFOA

We proposed a DNA sequence encoding design method based on the BFOA. This method utilized the replication characteristics of BFAO to obtain DNA sequences with certain intrinsic similarities, thereby achieving DNA sequences with similar melting temperatures. The key idea of this method is to optimize DNA sequence encoding design by leveraging the heuristic principles of bacterial foraging, hence the method is divided into four main stages: initialization, chemotaxis, replication, and dispersal. Each DNA sequence is mapped to a bacterium in the BFOA. Through these stages, inferior DNA sequences are eliminated while maintaining constant population size, yielding superior sequences.

The four main stages applied to DNA encoding design are as follows:

First stage: initialize some key parameters (see Table 1). For outputting a set of DNA sequences with low melting temperature, low mismatch probability, and high stability, seven critical initial parameters are required as inputs. These include the initial set of DNA sequences *D*_set_, the size of this initial set *D*_num_, the total number of replication cycles *N*_re_ that the DNA sequences can undergo, the number of base changes *N*_*C*_ that a DNA sequence can undergo within one replication cycle, the number of bases *E*_c_ that can be altered in a DNA sequence during a single base change event, the probability of DNA sequence elimination *p* within a replication cycle, and the number of DNA sequences *E*_*de*_ that are removed during each dispersal event.

Second stage: during each chemotaxis, each sequence in the set will alter several of its bases. Based on DNA encoding quality evaluation criteria, sequence quality is quantitatively assessed. If altered sequence scores exceed original scores, modified sequence enters the DNA set. Each round of chemotaxis generates a new DNA sequence set, replacing the previous set as the initial sequence set for subsequent rounds. This stage ensures that all DNA sequences are altered toward a more optimal direction.

Third stage: high-scoring DNA sequences are replicated while low-scoring sequences are discarded. Specifically, all sequences are ranked from high to low based on their scores; the top-ranked sequences are replicated and replace the lower-ranked ones. This replication process concludes the current round and initiates the next chemotaxis process. This semi-conservative replication method strongly eliminates inferior DNA sequences, preventing them from further participating in chemotaxis and degrading the overall quality of the sequence set (Table 1).

**Table 1:** Key initial parameters of the BFOA.

Parameters	Definition	Value
*N*_re_	The number of replication cycles (generations) in the BFOA algorithm	10
*NC*	The number of bases can be changed before each replication	20
*p*	Dispel the probability of an event	0.1
*E*_*de*_	The number of dispels per sequence	1
*D*_num_	The total number of sequences in the set	80
*E*_c_	Every time you change the number of bases	2

Fourth stage: to promote exploration and reduce premature convergence, a certain number of sequences are randomly eliminated with a given probability to increase population diversity, thereby reducing the risk of getting trapped in local optima. Although chemotaxis helps bacteria search within local regions and reproduction accelerates the search speed, for some complex optimization problems, bacteria can easily become trapped in local optima and fail to find the global optimum. To address this issue, the BFOA introduces elimination-dispersal behavior. This behavior simulates sudden environmental changes in nature, such as external disturbances or habitat alterations. In the algorithm, elimination-dispersal is implemented as randomly relocating a bacterium to an arbitrary position in the solution space with a certain probability. When a bacterium is selected for dispersal, it is removed from its current position and a new position is randomly generated elsewhere in the entire solution space. This random relocation mechanism effectively breaks the local optima trap that bacteria might fall into. When bacteria are at a local optimum, the surrounding environment may mislead them into believing that the global optimum has been found, thereby halting further exploration. Dispersal acts as a fresh start, enabling them to escape the current locally optimal region and explore other areas of the solution space where potentially better optima may exist. In this way, elimination-dispersal increases population diversity, enhances the global search capability of the algorithm, and allows the BFOA to more effectively search for the global optimum in complex solution spaces.

To enhance the response efficiency and reduce mismatch probability, the optimization goals of this section were low-decomposition temperature (TM) and low similarity (Sim). Double-stranded DNA reactions require temperature increase for strand separation, enabling base repair and replacement. Following single-strand formation, cooling enables double-helix reformation. High melting temperatures reduce reaction efficiency, as do large temperature variations among DNA strands. Sequence similarity also affects reaction quality; highly similar sequences may mis-hybridize, producing false-negative or incorrect structures. Low Tm requires both low average temperature and low variance. Low similarity requires minimal sequence similarity and measurement values.

In addition to optimizing the goals, this section considers seven constraints of basic biological characteristics: similarity (Sim), H-Measuer (Hm), continuity (Con), hairpin structure (Hair), relaxation temperature (TM), GC base content (GC), and specific sequence fragments. Sim and Hm measures are Hamming distance constraints. The Con and Hair are the constraints of the molecular secondary structure. The TM and GC content are thermodynamic stability constraints. The restricted nucleic acid-cutting sequence is a specific sequence fragment.

Sim is used to describe the similarities between alkaline base composition and arrangement of DNA sequence


(2) $$\mathrm{Sim}=\sum_{j=1}^{n}\min\begin{array}{c} j=1:n\&\&i\neq j\\ -m<k<m \end{array}[\mathrm{Ham}(X_{i},\mathrm{shift}(X_{j}^{k})]$$

where *n* represents the total number of DNA strands and *m* is the total number of bases in each DNA strand. For a wipeable sequence, when *k* > 0, $X_{i}$ slides to the right, otherwise it slides to the left. The Hamming distance between *a* and *b* is expressed as $\mathrm{Ham}(X_{i},\mathrm{shift}(X_{j}^{k})$. $\min\begin{array}{c} j=1:n\&\&i\neq j\\ -m<k<m \end{array}$ represents the minimum Hamming distance between $X_{j}$ and $X_{j}$ when $X_{i}$ slides from the left end to the right end.

*Hm* is used to describe the similarity between the base composition and arrangement of DNA sequences $X_{i}$ and $X_{j}^{c}$.$\;X_{j}^{c}$ is the complement of $X_{j}$


(3) $$Hm=\sum_{j=1}^{n}\min\begin{array}{c} j=1:n\\ -m<k<m \end{array}[\mathrm{Ham}(X_{i},\mathrm{shift}(X_{j}^{ck})]$$

Con is the continuous occurrence of an identical base in a DNA sequence. For DNA strand stability, the continuity constraint needs to be as low as possible.

Hair is a typical secondary structure due to self-folding single-stranded DNA molecules. A single-stranded DNA with a hairpin structure is essentially incapable of hybridizing to another single-stranded DNA. Therefore, the appearance of the hairpin structure should be avoided in the process of sequence design. The hairpin structure is defined as follows

$\mathrm{Hair}=\sum_{i=1}^{m}\sum_{P=\mathrm{Pmin}}^{n-\mathrm{Rmin}}\sum_{r=\mathrm{Rmin}}^{n=2p}\times T\times K$ (4)


(5) $$K=(\sum_{j=0}^{\mathrm{pinlen}(p,\;r,\;i)}\mathrm{bp}(X_{p+i-j},X_{p+i+j+1}),\frac{\mathrm{pinlen}(p,\;r,\;i)}{2})$$

(6) bp(Xp+i-j,Xp+i+j+1)={1 X1=X2c0 otherwise

(7) pinlen(p, r, i)=min⁡(p+i, n-p-i-r)

*p* is the length of the hairpin stem and *r* is the length of the hairpin loop. *Rmin* represents the minimum length that can form a hairpin loop, and *Pmin* is the minimum length that can form a hairpin stem.

The TM is defined as the temperature at which 50% of the base pairs in double-stranded DNA break the hydrogen bonds and form single-stranded DNA. A lower melting temperature means higher reaction efficiency. The melting temperature model in this article refers to the measured value of the nearest neighbor temperature model.

For the design of DNA sequence coding, we need to consider the relationship between equilibrium unwinding temperature and stability, to ensure low unwinding temperature and high stability. As a rule of thumb, in experiments, the proportion of GC bases is usually set to 50%.

After analyzing the coding target and coding constraints, it is anticipated that lower the unwinding temperature and the similarity of the DNA strands will be, better, the constraint of GC bases will be limited to 50%, and the continuity will be lower to ensure the stability of DNA strands and easy synthesis. Hair should be avoided as much as possible. The evaluation criterion is defined as the Eq. (8)


(8) S={STm+SSim+SHm+SCon GC=50%Hair=0 0 GC≠50%Hair=0

Tm, Sim, Hm and Con are assigned according to the weights of 40, 20, 20 and 20 points. For DNA sequences with lower unwinding temperature, similarity, H-measure and continuity, the score is higher; for DNA sequences with GC content not equal to 50% or hairpin structure not equal to 0, the score is 0.

The vital idea of this approach is to create aptamers of ASC-speck by exploiting the heuristic principle of BFOA, so the method is likewise divided into four main stages, namely initialization, chemotaxis, replication, and dispersal. With each DNA sequence mapped to each bacterium in the BFOA, after the above four main stages, some unfavorable DNA sequences can be eliminated under the condition that the total number remains unchanged, to obtain a better aptamer.

### 2.7 Docking analysis

To simulate the interaction between the generated aptamer and the ASC-speck biomarker, we used the HDOCK server (Yan *et al.* 2020) for ASC-speck aptamer docking. We selected four sequences with maximum and minimum scores for docking analysis.

### 2.8 Computational resources

All experiments were conducted on a server equipped with an Intel Xeon Gold 6248 CPU (2.5 GHz, 20 cores), an NVIDIA Tesla V100 GPU (32 GB memory), and 128 GB RAM. The operating system was Ubuntu 20.04 LTS, and the model was implemented using PyTorch 1.12.0 with CUDA 11.6.

## 3 Results

### 3.1 The proposed ASC2BF outperforms state-of-the-art methods

To comprehensively evaluate the predictive performance of ASC2BF, we compared it with several existing methods. Due to the limited number of relevant studies, we selected five representative approaches for comparison, as summarized in Table 2 (detailed algorithm descriptions are provided in Table 1, available as supplementary data at *Bioinformatics Advances* online). The first method (Heredia *et al.* 2021) employed an SVM classifier based on DNA aptamer sequences (238 samples) using 6-mer and sequence composition features, achieving an MCC of 0.896. The second approach (Muppirala *et al.* 2011) used a nearest neighbor algorithm with physicochemical features of target molecules (20 small molecule targets) and DNA/RNA aptamers (159 samples), yielding an MCC of 0.670; however, this method focused more on target-related electrostatic and chemical properties than on aptamer sequence characteristics. The third method (Li *et al.* 2014) applied a RF classifier to a larger dataset (725 DNA/RNA aptamers and 164 protein targets) using 1-mer and 2-mer features combined with protein physicochemical properties, obtaining an MCC of 0.461. The fourth approach employed an ensemble classifier on the same dataset, achieving an MCC of 0.478. The fifth method utilized a transformer-based model on the same dataset, reaching an MCC of 0.679. In comparison, our proposed ASC2BF, which integrates a deep multiscale residual network and is trained on 565 DNA aptamers, achieved the highest MCC of 0.915. These results demonstrate that ASC2BF significantly outperforms existing methods in distinguishing aptamers from non-aptamer sequences, while also possessing the unique capability of generating new aptamer sequences.

**Table 2:** Comparison of existing aptamer predictive studies.

Method	Aptamer dataset	Classifier	MCC
This study	DNA aptamers: 565	Deep multiscale ResNet	0.915
Heredia *et al.* (2021)	DNA aptamers 238	SVM	0.896
Muppirala *et al.* (2011)	DNA/RNA aptamers 159 small molecule targets 20	Nearest neighbors	0.67
Li *et al.* (2014)	DNA/RNA aptamers 725 protein target 164	Random forest	0.461
Yang *et al.* (2019)	DNA/RNA aptamers 725 protein target 164	Ensemble classifier	0.478
Shin *et al.* (2023)	DNA/RNA aptamers 725 protein target 164	Transformer	0.679

### 3.2 Multiscale feature design options are better suited to elucidate aptamer features

**Figure 2:**
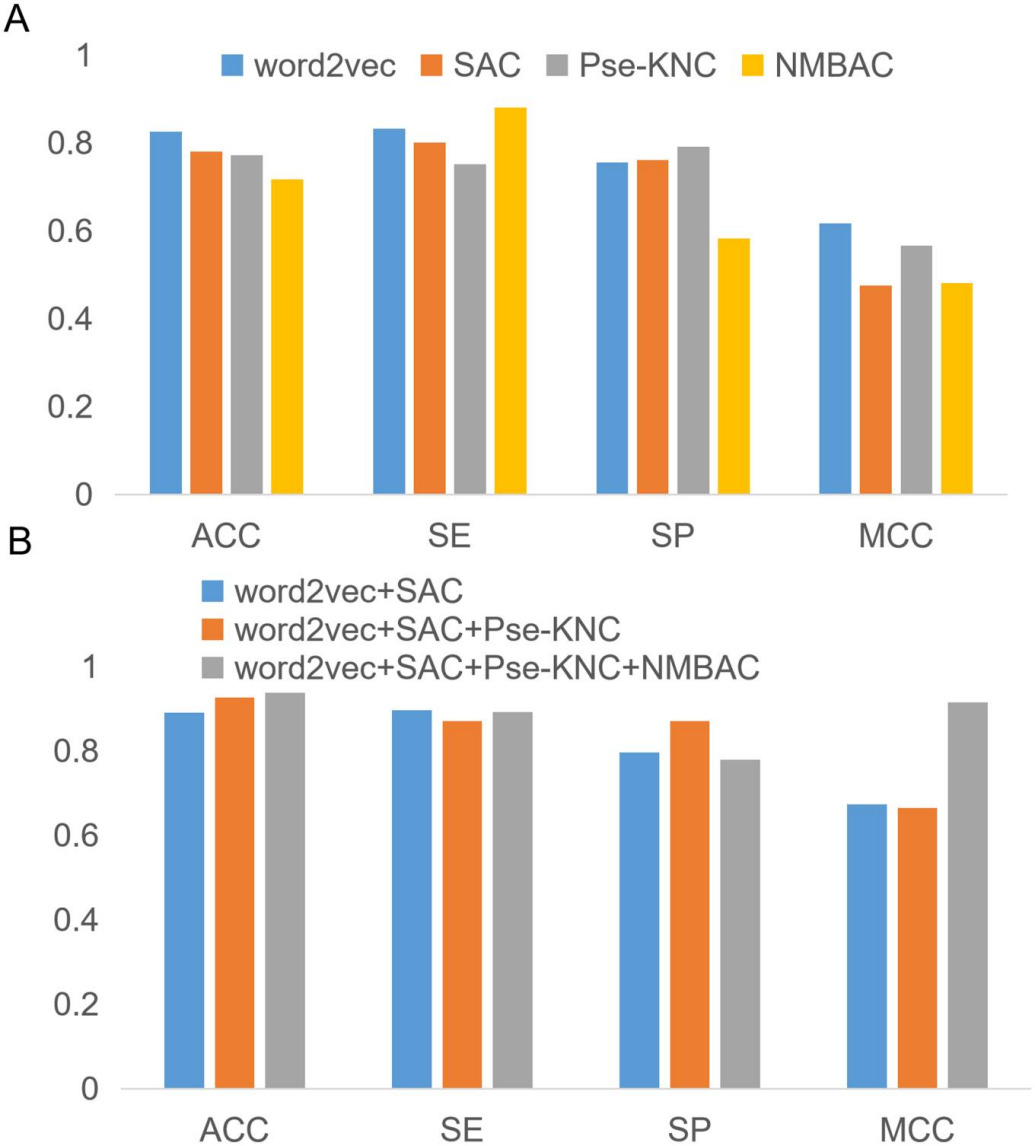
(A) Performance comparison of single features. (B) Performance comparison of multiscale features.

In ASC2BF, we proposed a multiscale information processing strategy for feature representation learning by using different features to represent different “biological words”. Therefore, we first validated how single-scale features affect the predictive performance of our model. The performance comparison results of single-scale features are shown in Fig. 2A. We can see that different feature expression methods do have their own advantages. No consistent results were observed. The feature representation of single-scale sequence model cannot capture the intrinsic features of adaptors adaptively and adequately. To solve this problem, we took features of different scales as model input and compare their performance, as shown in Fig. 2B. It can be found that multiscale features improve model performance. Among them, the average ACC of the multiscale feature fusion model on all data sets reached 0.937. This shows that features from different scales complement each other, which helps to learn feature representation comprehensively.

**Figure 3:**
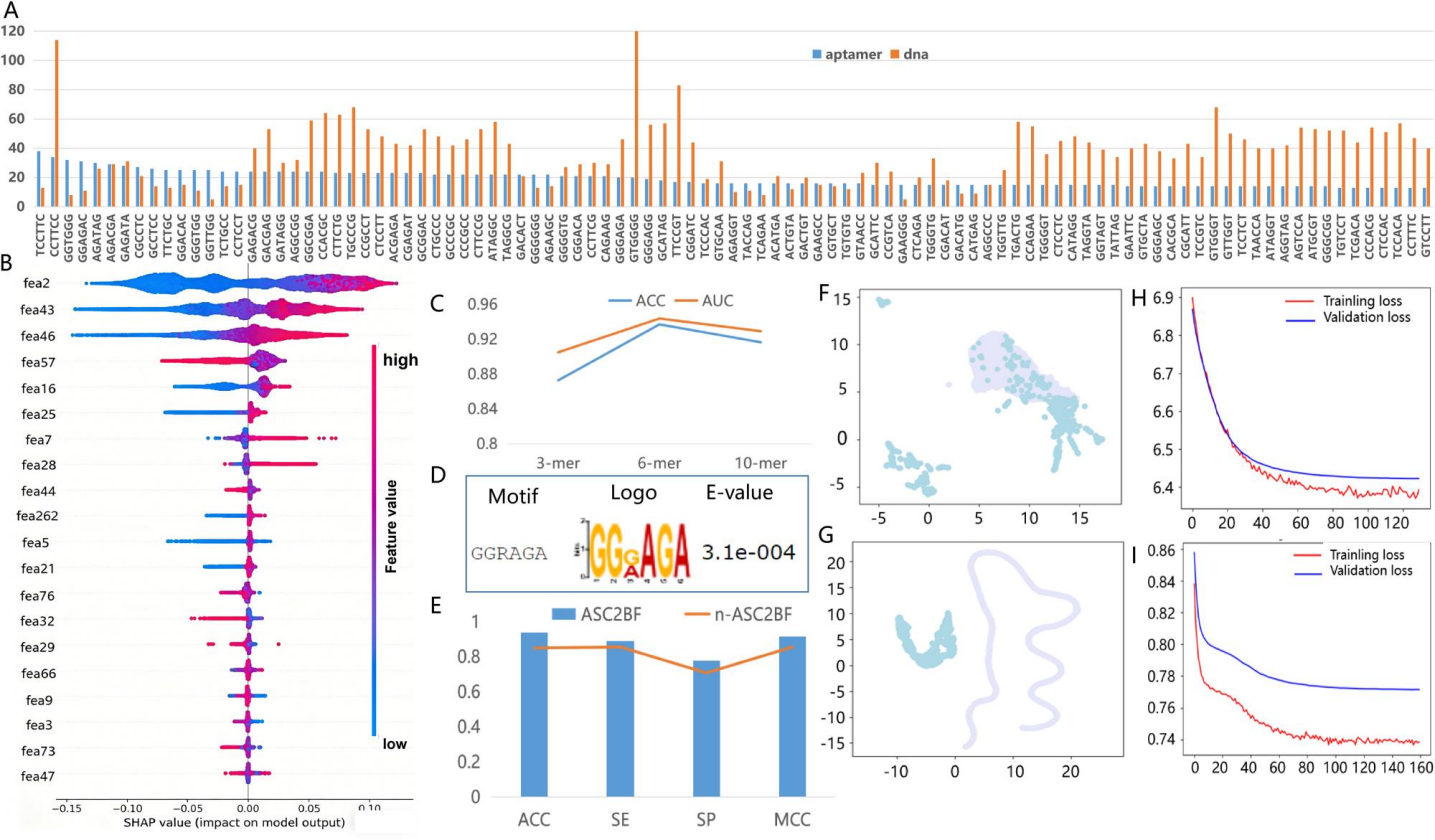
(A) Bars plot of selected 6-mers and their normalized distribution. (B) Summary plot for SHAP values. (C) ACC and AUC results for different *k* based on word2vec. (D) Motifs of aptamers. (E) Performance of models with and without MRN. (F, G) T-SNE visualization scatter plots of pre-training (F) and post-training (G) feature representations. (H, I) Training and validation loss curves of ASC2BF with MRN (H) and without MRN (I). Solid lines: training loss; dashed lines: validation loss.

We then analyzed the distinctive characteristics of DNA aptamers and protein-bound DNA (as Fig. I, available as supplementary data at *Bioinformatics Advances* online). The density map visualizes the relationship between these features in the data set. Where, the diagonal line represents the distribution of a single feature. The density maps of purine and pyrimidine distributions show thin peaks. For other features, aptamers are distributed in thin ends, while DNA features are distributed in Gauss. These diagrams also show that, irrespective of other factors, these single features are not good enough to distinguish aptamers from DNA. According to previous studies, the 6-mer feature plays a vital role in the aptamer, so we further analyzed the difference in the distribution of the 6-mer feature between the aptamer and the protein-binding DNA and the effect on the model performance. Figure 3A plots the observed occurrence of 6-mer. Each oligonucleotide was calculated and normalized. As can be seen from Fig. 3A, the distribution of 6-mers varies greatly from DNA to aptamers, and 6-mers with high GG content appear more frequently in aptamers.

Then, we used SHAP (Mokhtari *et al.* 2019) to calculate the importance value under the 6-mer feature encoding scheme, and counted the absolute mean distribution of the SHAP values of the top 20 features to determine which features were most important for aptamer recognition. The results are shown in the honeycomb plot in Fig. 3B. Each point in the graph represents a real sample. For each set of samples, the larger the eigenvalue, the redder the color of the point. The more points with the same SHAP value, the larger the cross-sectional area of the honeycomb and the thicker it appears. The fea2 feature has the greatest influence, and the 6mer is CCTTCC; the fea43 and fea46 are next, and the 6-mer is GTGGGG and TTCCGT, respectively.

After that, we analyzed the performance of ASC2BF of *k*-mer under different *k* values under the word2vec algorithm. Figure 3C counts the ACC and AUC values of the ASC2BF model when *k* is 3, 6, and 10. It was found that when *k* = 6, the ACC and AUC values of ASC2BF are the highest, which is consistent with the speculation that 6-mers may have important functional or structural significance in these aptamers.

### 3.3 Our ASC2BF reveals a sequence pattern in the aptamer

The results so far underscore the performance of ASC2BF. To delve deeper into the post-prediction causes, we applied interpretative analysis methods to analyze the key input sequence contents that are important for prediction fitness. Based on *k* = 6, we summarized and examined the consensus themes that play a vital role in the ASC2BF model and how they relate to word2vec. We further analyzed aptamer sequence motifs using the probability-based motif visualization tool STREME (https://meme-suite.org/meme/tools/streme). The width of the conservative motif is set to 6, consistent with the word2vec feature encoding word length.

The most frequently occurring 6-mer in word2vec are GGGAGA, GGGCCA, CCCCCC, TTGCAC, and GGCGCC. The motif obtained by using STREME is shown in Fig. 3D. The resulting motif is very similar to the highest frequency 6-mer. The results also show that the sequence pattern of the aptamer is ordered, and the word embedding model seems to learn the subtle sequence context from the semantics and syntax, thus improving the recognition performance.

Of the five aggregates obtained, four were found to be structures in the PDB (as Fig. II, available as supplementary data at *Bioinformatics Advances* online) and involved in protein binding. Six-mers are short ring elements in DNA sequences that are both conserved and vary between species because of their functional importance. Six-mers may be a core fragment of binding sites or a regulatory element involved in protein binding. Therefore, we hypothesize that 6-mers in aptamers have biological or structural importance. To study the biological importance of 6-mers, we compared the first four 6-mers with the aptamer structures included in the PDB database (as Table 2, available as supplementary data at *Bioinformatics Advances* online). Although 6-mers may be functionally or structurally important in these aptamers, the conclusion that 6-mers always has biological significance needs further study and validation.

### 3.4 Multiscale residual network enhances the deep feature learning ability of ASC2BF

MRN is a core component of ASC2BF for learning biological context semantics. To evaluate its effectiveness, we compared the full ASC2BF model with a variant that excludes the MRN module (denoted as n-ASC2BF). As shown in Fig. 3E, ASC2BF consistently outperforms n-ASC2BF in terms of both ACC and MCC, indicating that the multiscale residual architecture captures meaningful sequence features that improve aptamer prediction.

To further visualize the discriminative power of MRN, we used t-SNE to visualize the feature distributions before and after training (Fig. 3F and G). Before training (Fig. 3F), aptamer and non-aptamer samples are interleaved with no clear boundary. After training (Fig. 3G), the two classes form two compact, well-separated clusters. This indicates that MRN effectively transforms raw sequence encodings into a semantically meaningful feature space where aptamers are distinguishable from non-aptamers.

We also analyzed the training and validation loss curves (Fig. 3H and I). The ASC2BF model achieves lower validation loss and maintains stable training loss, whereas n-ASC2BF exhibits a gradual increase in validation loss after initial convergence—a typical sign of overfitting. This suggests that the MRN not only improves prediction accuracy but also enhances model robustness and generalization ability.

### 3.5 Our model ASC2BF is good for improving imbalanced datasets

Subsequently, to address the data imbalance, we implemented OHEM, uncertainty weighting (UW) and focal loss on the optimized model and tested their performance. Then the improved AUC value is obtained according to the optimized model (Table 3). Both OHEM and UW are good for partial remodeling. OHEM’s prediction improved the AUC score by 0.08, while UW’s prediction improved by 0.06. After we combined the two, it helped improve the AUC score by 0.0145.

**Table 3:** Comparison of imbalance handling methods.

Method	ACC	SE	SP	MCC	AUC
SMOTE	0.852	0.852	0.697	0.938	0.8691
UW	0.831	0.831	0.706	0.948	0.8725
Focal Loss	0.766	0.766	0.643	0.949	0.8751
OHEM	0.937	0.891	0.779	0.882	0.9787

### 3.6 Comparison with other classifiers

To further evaluate the predictive power of ASC2BF, we compared its performance against several widely used machine learning classifiers, including RF, LibSVM, Naïve Bayes (Bayes), and AdaBoost, using the same dataset and experimental setup. The comparison results are summarized in Table 4.

**Table 4:** Comparison of other classifiers.

Method	ACC	SE	SP	MCC	AUC
ASC2BF	0.852	0.852	0.698	0.938	0.869
RF	0.811	0.815	0.827	0.781	0.826
LibSVM	0.590	0.774	0.738	0.451	0.651
Bayes	0.696	0.733	0.611	0.311	0.711
AdaBoost	0.843	0.548	0.696	0.409	0.760

As shown, ASC2BF achieves the highest accuracy (ACC = 0.8524), Matthew’s correlation coefficient (MCC = 0.9381), and area under the ROC curve (AUC = 0.8691) among all compared methods. Notably, ASC2BF also exhibits a balanced sensitivity (SE = 0.8524) and specificity (SP = 0.6969). In contrast, RF achieves moderate performance (ACC = 0.8109, MCC = 0.7811, AUC = 0.8257), while LibSVM and Bayes yield considerably lower MCC values (0.4507 and 0.3111, respectively), indicating poor classification reliability. AdaBoost shows competitive accuracy (0.8433) but suffers from low sensitivity (0.5481) and a low MCC (0.4090). These results demonstrate that ASC2BF, which integrates deep multiscale residual networks with advanced feature learning, significantly outperforms traditional classifiers in distinguishing aptamers from non-aptamer DNA sequences.

### 3.7 Performance comparison with other coding methods

To demonstrate the sequence encoding generation capability of our proposed framework, this method is compared with the encoding algorithm NGIWO (Zhu *et al.* 2022) genetic algorithm (GA) and particle swarm optimization (PSO). For each method, we generated four sequences of 12 nucleotides and evaluated them on six criteria: continuity (Con), hairpin structure (Hair), H‑measure (Hm), similarity (Sim), melting temperature (Tm), and GC content. The results are summarized in Table 5.

**Table 5:** Performance comparison with other coding methods.

Method	Sequence	Con	Hair	Hm	Sim	Tem	GC	Time (h)
ASC2BF	TCGCCTGGTCAT	0	0	68	53	49.7	50	3.9
	CGCCTGAAGAGG	0	0	63	50	48.55	50	
	GGGCGGATAATT	0	0	64	52	50.65	50	
	GTACCAGTACAC	0	0	63	50	52.08	50	
NGIWO	GATGGATTTACC	0	0	43	58	47.68	50	5.8
	GTTCAATCGCCT	0	0	37	58	46.94	50	
	GCTACCTCTTCC	0	0	45	57	49.10	50	
	GAATCAATGGCG	0	0	52	56	51.13	50	
GA	AGAGTACGTCAG	0	0	60	49	63.53	50	3.7
	TGCTGTAGATCG	0	0	57	45	66.05	50	
	CTACTACGAGTC	0	0	57	46	61.63	50	
	GTGAGAGCTCAG	0	0	58	52	62.29	50	
PSO	ACACACACTCAC	9	4	51	45	60.72	45	4.1
	CATACGTGAGTG	0	0	50	44	61.52	45	
	GCAGATTCCCGG	0	3	51	44	59.84	45	
	CTGGAAGCGTTT	9	7	50	43	62.04	45	

From Table 5, the following observations can be made: Con & Hair: ASC2BF, NGIWO, and GA all achieve zero for both metrics, indicating strict satisfaction of structural constraints. PSO yields poor values (Con up to 9, Hair up to 7), indicating inferior sequence quality. GC content: ASC2BF, NGIWO, and GA maintain the optimal 50% GC, whereas PSO gives only 45%, deviating from the target. Sim & Hm: ASC2BF shows slightly higher Hm (63–68) and Sim (50–53) than NGIWO (Hm 37–52, Sim 56–58), but these values remain within acceptable limits and reflect a tradeoff between cross-hybridization avoidance and sequence diversity. Tm: ASC2BF produces the lowest and most consistent melting temperatures (range 48.55–52.08, average: 50.25), outperforming GA (61.63–66.05) and PSO (59.84–62.04). NGIWO gives slightly lower Tm values (46.94–51.13) but with greater variance, indicating less uniform melting behavior. In summary, ASC2BF demonstrates superior overall performance by strictly satisfying all biological constraints (Con = 0, Hair = 0, GC = 50%) while producing sequences with low, consistent melting temperatures. Although its similarity metrics are slightly higher than those of NGIWO, the tradeoff is acceptable given the significant gains in thermodynamic stability and structural purity.

In addition to sequence quality, we compared the computational efficiency of each method under identical hardware conditions (see Section 2.8). ASC2BF required approximately 3.9 h to generate 1000 candidate sequences (12 nt each) using BFOA with full constraint evaluation, which is faster than NGIWO (5.8 h) and PSO (4.1 h), and comparable to GA (3.7 h), while achieving better sequence quality (Table 5).

### 3.8 Selection of ASC-speck binding aptamer using BFOA

To investigate aptamers that can bind to ASC-speck proteins, we used the predicted sequences as the initial population of BFOA to generate new sequences (Table 6). According to the fitness value of BFOA fitness function, 1000 top fitness sequences were selected. The experimental results show that the 438 generation has the highest binding potential and the highest score, while the 149 generation has the lowest average score. The binding probability of the 438 generation sequence is 3–6 times higher than that of the *in vivo* initial DNA or artificially generated oligonucleotide progenitor cells. For further study, DNA aptamers with the highest and lowest scores were selected as candidates for docking and dynamic molecular simulation analysis.

**Table 6:** Initial population of BFOA.

Row	Sequence	Score
1	TACCAGTGCGATGCTCAGTAACTTTGAAGGAAAGGCTACAAACTCTTCCTGACGCATTCGGTTGAC	3.87
2	AACGTGGGAGGGCGGTGGTGTTGAA	0.92
3	ATACGGGAGCCAACACCATCCCTCTTAGGATACAAAGCCAAACTGAGCCCGTGCAGAGCAGGTGTGACGGAT	0.74
4	CCAGTCTCCCGTTTACCGCGCCTACACATGTCTGAATGCC	0.24
5	CACGTCCATCTCTGCAGTCGGGTAGTTAAACCGACCTTCAGACATAGTAAGGGG	0.60
6	GGGAACGCACCGATCGCAGGTTTCCC	0.13
7	GGAGACCGTACCATCTGTTCGTGGAAGCGCTTTGCTCGTCCATTAGCCTTGTGCTCGTGC	0.18

**Figure 4:**
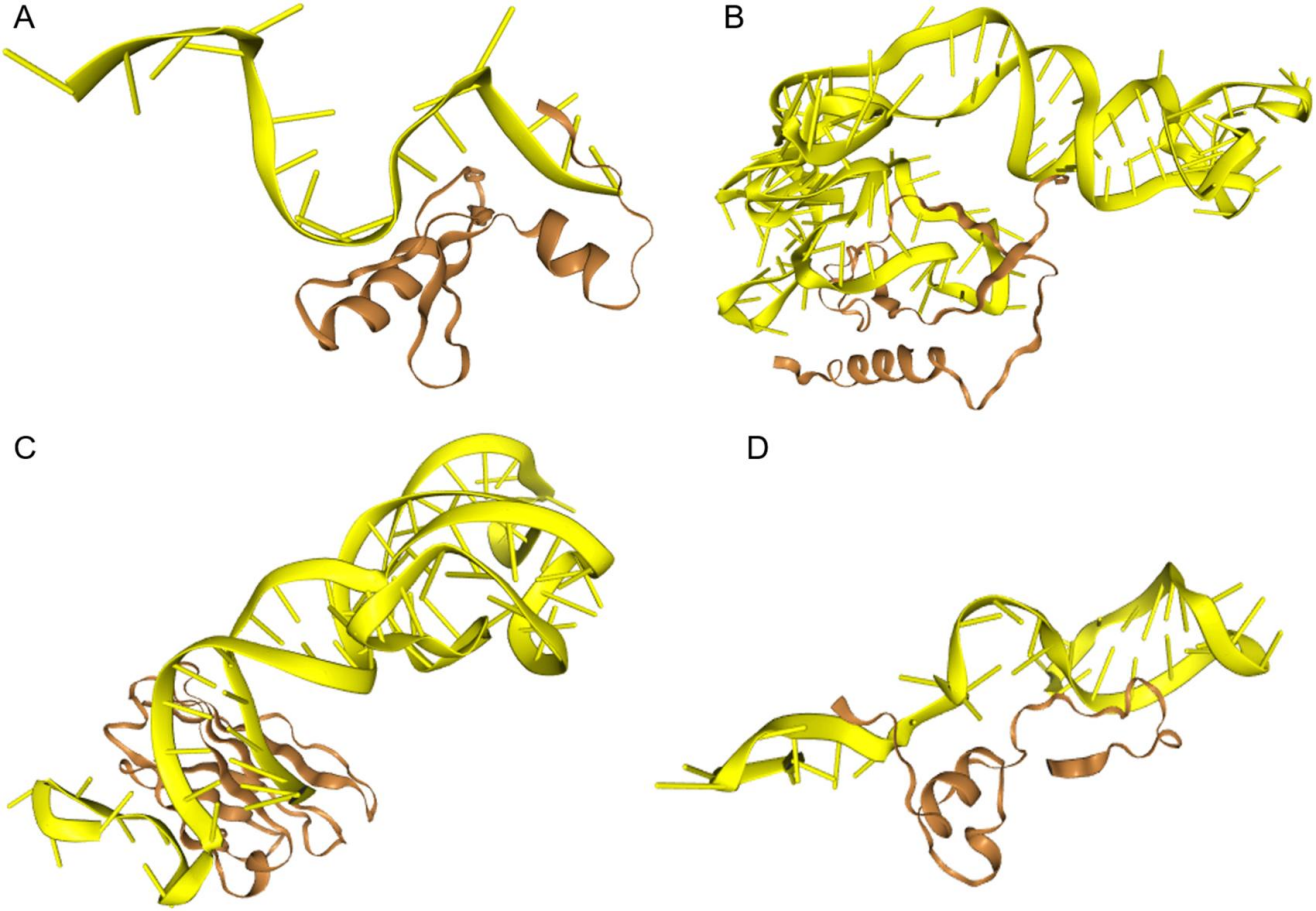
3D illustration of docking results for aptamers and ASC-speck.

According to S (Eq. (8)), we select four sequences generated by BFOA algorithm: the first two sequences with high scores and the last two sequences with low scores (denoted as high1, high2, low1, low2, respectively) to dock with ASC-speck. In each docking simulation, the first docking model with the highest energy score will be selected for molecular simulation. Figure 4 shows the docking results with aptamers and ASC-speck. The docking energy score is shown in Table 7. It can be found that the docking energy fraction of high2 is greater than low2, low2 is greater than high1, and high1 is greater than low1. The negative docking energy score was the highest, indicating that the aptamer had a good affinity for the protein.

**Table 7:** Docking energy score of selected sequences.

Number	Sequence	Name	GS1	GS2	Docking score
1	TCGCCTGGTCATGGGCGTGCGACGGGCGCCCAT	High1	6.25	21.25	−221.50
2	CGCCTGAAGAGGTAGGGTGGGTGCTAGGCGTACCACGGTT	Low1	0.5	16.6	−173.70
3	GGGCGGATAATTAGCGGGCG	High2	6.5	26.8	−257.65
4	GTACCAGTACACTCAATTGGGTTTATTCCATGCAAACCCAATAGTAAAGTGGGTGCTGGATCCTG	Low2	0.55	22.5	−223.40

## 4 Conclusion

In this study, we developed a DL-based framework, ASC2BF, for classifying DNA sequences as aptamers or non-aptamers. The proposed method achieves satisfactory predictive performance and demonstrates the ability to learn semantic information from sequences. Using ASC2BF, we generated aptamer sequences targeting ASC-speck proteins, and docking results confirmed the effectiveness of our approach. This work provides a promising computational tool for aptamer selection across a wide range of biomarkers.

Despite these encouraging results, several limitations remain. First, although our heuristic DNA coding design theoretically reduces melting temperature and sequence similarity, practical biochemical implementation may encounter unforeseen challenges. Collaboration with experimental biologists is therefore essential to validate and refine the coding strategies. Second, the current framework does not account for differences in binding strength among aptamers or variations across different target types. Third, and most importantly, all results are currently based on in silico analyses; experimental validation is required to confirm the actual binding affinity and specificity of the generated aptamers.

In future work, we plan to (i) actively seek cooperation with laboratories and integrate experimental assays (e.g. surface plasmon resonance) to validate the top-ranking aptamer candidates, (ii) extend the framework to other protein targets by transfer learning, and (iii) adapt the constraint settings to accommodate specific practical applications. We believe that combining deep learning-guided design with biological experimentation will ultimately advance aptamer discovery for therapeutic and diagnostic purposes.

## Supplementary Material

vbag168_Supplementary_Data

## Data Availability

The data underlying this article are available in https://github.com/nmt315320/aptamer.git.

## References

[vbag168-B1] Adachi T , Nakamura Y. Aptamers: a review of their chemical properties and modifications for therapeutic application. Molecules 2019;24:4229. 31766318 10.3390/molecules24234229 PMC6930564

[vbag168-B2] Ahmad NA , Mohamed Zulkifli R, Hussin H et al In silico approach for Post-SELEX DNA aptamers: a mini-review. J Mol Graph Model 2021;105:107872. 33765525 10.1016/j.jmgm.2021.107872

[vbag168-B3] Bege M , Ghanem Kattoub R, Borbás A. The 20th anniversary of Pegaptanib (MacugenTM), the first approved aptamer medicine: history, recent advances and future prospects of aptamers in therapy. Pharmaceutics 2025;17:394. 40143057 10.3390/pharmaceutics17030394 PMC11944999

[vbag168-B4] Berezovski M , Drabovich A, Krylova SM et al Nonequilibrium capillary electrophoresis of equilibrium mixtures: a universal tool for development of aptamers. J Am Chem Soc 2005;127:3165–71. 15740156 10.1021/ja042394q

[vbag168-B5] Chang YM , Donovan MJ, Tan W. Using aptamers for cancer biomarker discovery. J Nucleic Acids 2013;2013:817350. 23401749 10.1155/2013/817350 PMC3562578

[vbag168-B6] Chaturvedi S , Naseem Z, El-Khamisy SF et al Nanomedicines targeting the inflammasome as a promising therapeutic approach for cell senescence. Semin Cancer Biol 2022;86:46–53. 10.1016/j.semcancer.2022.08.008 36030027

[vbag168-B7] Chawla NV , Bowyer KW, Hall LO et al SMOTE: synthetic minority over-sampling technique. jair 2002;16:321–57.

[vbag168-B8] Chen Z , Hu L, Zhang B-T et al Artificial intelligence in aptamer–target binding prediction. Int J Mol Sci 2021;22:3605. 33808496 10.3390/ijms22073605 PMC8038094

[vbag168-B9] Church KW. Word2Vec. Nat Lang Eng 2017;23:155–62.

[vbag168-B10] Chushak Y , Stone MO. In silico selection of RNA aptamers. Nucleic Acids Res 2009;37:e87. 19465396 10.1093/nar/gkp408 PMC2709588

[vbag168-B11] Cruz-Toledo J , McKeague M, Zhang X et al Aptamer base: a collaborative knowledge base to describe aptamers and SELEX experiments. Database 2012;2012 10.1093/database/bas006 PMC3308162 22434840

[vbag168-B12] Cucchiarini A , Dobrovolná M, Brázda V et al Analysis of quadruplex propensity of aptamer sequences. Nucleic Acids Res 2025;53:gkaf424. 40377215 10.1093/nar/gkaf424 PMC12082452

[vbag168-B13] Emami N , Ferdousi R. AptaNet as a deep learning approach for aptamer–protein interaction prediction. Sci Rep 2021;11:6074. 33727685 10.1038/s41598-021-85629-0 PMC7971039

[vbag168-B14] Emami N , Pakchin PS, Ferdousi R. Computational predictive approaches for interaction and structure of aptamers. J Theor Biol 2020;497:110268. 32311376 10.1016/j.jtbi.2020.110268

[vbag168-B15] Englert D , Burger E-M, Grün F et al Fast-exchanging spirocyclic rhodamine probes for aptamer-based super-resolution RNA imaging. Nat Commun 2023;14:3879. 37391423 10.1038/s41467-023-39611-1 PMC10313827

[vbag168-B16] Groff K , Brown J, Clippinger AJ. Modern affinity reagents: recombinant antibodies and aptamers. Biotechnol Adv 2015;33:1787–98. 26482034 10.1016/j.biotechadv.2015.10.004

[vbag168-B17] Gupta T , Sharma P, Malik S et al AIoptamer: artificial intelligence-driven aptamer optimization pipeline for targeted therapeutics in healthcare. Mol Pharm 2025;22:4076–90. 40531093 10.1021/acs.molpharmaceut.5c00343

[vbag168-B18] Heredia FL , Roche-Lima A, Parés-Matos EI. A novel artificial intelligence-based approach for identification of deoxynucleotide aptamers. PLoS Comput Biol 2021;17:e1009247. 34343165 10.1371/journal.pcbi.1009247 PMC8362955

[vbag168-B19] Hung L-Y , Fu C-Y, Wang C-H et al Microfluidic platforms for rapid screening of cancer affinity reagents by using tissue samples. Biomicrofluidics 2018;12:054108. 30344835 10.1063/1.5050451 PMC6170194

[vbag168-B20] Jain P , Tiwari A, Som T. Intuitionistic fuzzy rough set model based on *k*-means and its application to enhance prediction of aptamer–protein interacting pairs. J Ambient Intell Human Comput 2024;15:3575–86.

[vbag168-B21] Jayasena SD. Aptamers: an emerging class of molecules that rival antibodies in diagnostics. Clin Chem 1999;45:1628–50. 10471678

[vbag168-B22] Jenster L, Ribeiro LS, Franklin BS et al Measuring NLR oligomerization II: detection of ASC speck formation by confocal microscopy and immunofluorescence. In: NLR Proteins: Methods and Protocols. New York, NY: Springer US, 2023, 73–92. 10.1007/978-1-0716-3350-2_5 37578716

[vbag168-B23] Knight CG , Platt M, Rowe W et al Array-based evolution of DNA aptamers allows modelling of an explicit sequence-fitness landscape. Nucleic Acids Res 2009;37:e6. 19029139 10.1093/nar/gkn899 PMC2615635

[vbag168-B24] Kohlberger M , Gadermaier G. SELEX: critical factors and optimization strategies for successful aptamer selection. Biotechnol Appl Biochem 2022;69:1771–92. 34427974 10.1002/bab.2244 PMC9788027

[vbag168-B25] Li B-Q , Zhang Y-C, Huang G-H et al Prediction of aptamer–target interacting pairs with pseudo-amino acid composition. PLoS One 2014;9:e86729. 24466214 10.1371/journal.pone.0086729 PMC3899287

[vbag168-B26] Li J , Ma X, Li X et al PPAI: a web server for predicting protein-aptamer interactions. BMC Bioinformatics 2020;21:236. 32517696 10.1186/s12859-020-03574-7 PMC7285591

[vbag168-B27] Mokhtari KE , Higdon BP, Başar A. Interpreting financial time series with SHAP values. In: *Proceedings of the 29th Annual International Conference on Computer Science and Software Engineering*. Markham, ON, 2019, 166–72.

[vbag168-B28] Muppirala UK , Honavar VG, Dobbs D. Predicting RNA–protein interactions using only sequence information. BMC Bioinformatics 2011;12:489. 22192482 10.1186/1471-2105-12-489 PMC3322362

[vbag168-B29] Niu M , Lin Y, Zou Q. sgRNACNN: identifying sgRNA on-target activity in four crops using ensembles of convolutional neural networks. Plant Mol Biol 2021;105:483–95. 33385273 10.1007/s11103-020-01102-y

[vbag168-B30] Niu M , Zou Q, Lin C. CRBPDL: identification of circRNA-RBP interaction sites using an ensemble neural network approach. PLoS Comput Biol 2022;18:e1009798. 35051187 10.1371/journal.pcbi.1009798 PMC8806072

[vbag168-B31] Preeti K , Jain N, Sharma SK. Advances in SELEX methods. In: Aptasensors for Food Safety. CRC Press, 2024, 93–105.

[vbag168-B32] Ren J , Yao Y. DNA computing sequence design based on bacterial foraging algorithm. In: *Proceedings of the 2018 5th International Conference on Bioinformatics Research and Applications*. New York, NY, 2018, 1–7.

[vbag168-B33] Ruiz-Ciancio D , Veeramani S, Singh R et al AptamerRunner: an accessible aptamer structure prediction and clustering algorithm for visualization of selected aptamers. Mol Ther Nucleic Acids 2024;35:102358. 39507401 10.1016/j.omtn.2024.102358 PMC11539416

[vbag168-B34] Shin I , Kang K, Kim J et al AptaTrans: a deep neural network for predicting aptamer–protein interaction using pretrained encoders. BMC Bioinformatics 2023;24:447. 38012571 10.1186/s12859-023-05577-6 PMC10680337

[vbag168-B35] Shrivastava A , Gupta A, Girshick R. Training region-based object detectors with online hard example mining. In: *Proceedings of the IEEE Conference on Computer Vision and Pattern Recognition*. Las Vegas, NV, 2016, 761–9.

[vbag168-B36] Soundararajan S , Chen W, Spicer EK et al The nucleolin targeting aptamer AS1411 destabilizes Bcl-2 messenger RNA in human breast cancer cells. Cancer Res 2008;68:2358–65. 18381443 10.1158/0008-5472.CAN-07-5723

[vbag168-B37] Stangherlin S , Lui N, Lee JH et al Aptamer-based biosensors: from SELEX to biomedical diagnostics. TrAC Trends Anal Chem 2025;191:118349.

[vbag168-B38] Ștefan G , Hosu O, De Wael K et al Aptamers in biomedicine: selection strategies and recent advances. Electrochim Acta 2021;376:137994.

[vbag168-B39] Stutz A et al ASC speck formation as a readout for inflammasome activation. In: The Inflammasome: Methods and Protocols. Totowa, NJ: Humana Press, 2013, 91–101. 10.1007/978-1-62703-523-1_8 23852599

[vbag168-B40] Tuerk C, Gold L. Systematic evolution of ligands by exponential enrichment: RNA ligands to bacteriophage T4 DNA polymerase. Science 1990;249:505–10. 2200121 10.1126/science.2200121

[vbag168-B41] Vandghanooni S , Eskandani M, Barar J et al Bispecific therapeutic aptamers for targeted therapy of cancer: a review on cellular perspective. J Mol Med (Berl) 2018;96:885–902. 30056527 10.1007/s00109-018-1669-y

[vbag168-B42] Wang Y , Zhai Y, Ding Y et al SBSM-Pro: support bio-sequence machine for proteins. Sci China Inf Sci 2024;67:212106.

[vbag168-B43] Wang Z , Liu Z, Zhang W et al AptaDiff: de novo design and optimization of aptamers based on diffusion models. Brief Bioinform 2024;25:bbae517. 39431516 10.1093/bib/bbae517 PMC11491854

[vbag168-B44] Yan Y , Tao H, He J et al The HDOCK server for integrated protein–protein docking. Nat Protoc 2020;15:1829–52. 32269383 10.1038/s41596-020-0312-x

[vbag168-B45] Yang Q , Jia C, Li T. Prediction of aptamer–protein interacting pairs based on sparse autoencoder feature extraction and an ensemble classifier. Math Biosci 2019;311:103–8. 30880100 10.1016/j.mbs.2019.01.009

[vbag168-B46] Yang X , Chan CH, Yao S et al DeepAptamer: advancing high-affinity aptamer discovery with a hybrid deep learning model. Mol Ther Nucleic Acids 2025;36:102436. 39897584 10.1016/j.omtn.2024.102436 PMC11787022

[vbag168-B47] Zhang L , Zhang C, Gao R et al Prediction of aptamer–protein interacting pairs using an ensemble classifier in combination with various protein sequence attributes. BMC Bioinformatics 2016;17:225. 27245069 10.1186/s12859-016-1087-5 PMC4888498

[vbag168-B48] Zhou J , Rossi J. Aptamers as targeted therapeutics: current potential and challenges. Nat Rev Drug Discov 2017;16:181–202. 27807347 10.1038/nrd.2016.199 PMC5700751

[vbag168-B49] Zhu D , Huang Z, Liao S et al Improved bare bones particle swarm optimization for DNA sequence design. IEEE Trans Nanobioscience 2023;22:603–13. 36350858 10.1109/TNB.2022.3220795

