# Supplementary material for: Interpretable prediction and generation of ASC-speck aptamers using multiscale deep biological learning models
Source: Bioinform Adv. 2026 Jun 13;6(1):vbag168. doi: 10.1093/bioadv/vbag168 (PMC13308713; doi:10.1093/bioadv/vbag168)
Supplement: vbag168_Supplementary_Data [file vbag168_supplementary_data.docx]

**Supporting Information:**

**Text .S1.** BFOAS1, More detailed descriptions of the algorithm BFOA.

**Figure SI.** A-H. Plot of the DNA vs. aptamer features.

**Figure SII.** Structures of elucidated aptamers with their sequences. (A) 3HXO;(B)7Z29;(C) 4QJU;(D)5TXN.

**Table SI.** Comparison of existing aptamer predictive studies.

**Table SII.** Identification of the top 6-mers on elucidated aptamer structures.

Text .S1. BFOAS1, More detailed descriptions of the algorithm BFOA.

In addition to optimizing the goals, this section considers seven constraints of basic biological characteristics: similarity (Sim), H-Measuer (Hm), continuity (Con), hairpin structure (Hair), relaxation temperature (TM), GC base content (GC) and specific sequence fragments. Sim and Hm measures are Hamming distance constraints. The Con and Hair are the constraints of the molecular secondary structure. The TM and GC content are thermodynamic stability constraints. The restricted nucleic acid -cutting sequence is a specific sequence fragment.

Sim is used to describe the similarities between alkaline base composition and arrangement of DNA sequence.

$Sim=\sum_{j=1}^{n} min\begin{matrix} j=1:n\&\&i\neq j \\ -m<k<m \end{matrix}[Ham(X_{i},shift(X_{j}^{k})]$ (1)

where n represents the total number of DNA strands and m is the total number of bases in each DNA strand. For a wipeable sequence, when k>0, $X_{i}$ slides to the right, otherwise it slides to the left. The Hamming distance between a and b is expressed as $Ham(X_{i},shift(X_{j}^{k})$. $min\begin{matrix} j=1:n\&\&i\neq j \\ -m<k<m \end{matrix}$ represents the minimum Hamming distance between $X_{j}$ and $X_{j}$ when $X_{i}$ slides from the left end to the right end.

Hm is used to describe the similarity between the base composition and arrangement of DNA sequences $X_{i}$ and $X_{j}^{c}$.$X_{j}^{c}$ is the complement of $X_{j}$.

$Hm=\sum_{j=1}^{n} min\begin{matrix} j=1:n \\ -m<k<m \end{matrix}[Ham(X_{i},shift(X_{j}^{ck})]$ (2)

Con is the continuous occurrence of an identical base in a DNA sequence. For DNA strand stability, the continuity constraint needs to be as low as possible.

Hair is a typical secondary structure due to self-folding single-stranded DNA molecules. A single-stranded DNA with a hairpin structure is essentially incapable of hybridizing to another single-stranded DNA. Therefore, the appearance of the hairpin structure should be avoided in the process of sequence design. The hairpin structure is defined as follows.

$Hair=\sum_{i=1}^{m} \sum_{P=Pmin}^{n-Rmin} \sum_{r=Rmin}^{n=2p} \times T\times K$ (3)

$K=(\sum_{j=0}^{pinlen\left( p,r,i \right)} bp\left( X_{p+i-j},X_{p+i+j+1} \right),\frac{pinlen(p,r,i)}{2})$ (4)

$bp\left( X_{p+i-j},X_{p+i+j+1} \right)=\left\{ \begin{aligned} 1 X_{1}=X_{2}^{c} \\ 0 otherwise \end{aligned} \right.$ (5)

$pinlen\left( p,r,i \right)=min(p+i,n-p-i-r)$ (6)

𝑝 is the length of the hairpin stem, and 𝑟 is the length of the hairpin loop. 𝑅𝑚𝑖𝑛 represents the minimum length that can form a hairpin loop, and 𝑃𝑚𝑖𝑛 is the minimum length that can form a hairpin stem.

The TM is defined as the temperature at which 50% of the base pairs in double-stranded DNA break the hydrogen bonds and form single-stranded DNA. A lower de rotation temperature means higher reaction efficiency. The de rotation temperature model in this paper refers to the measured value of the nearest neighbor temperature model.

For the design of DNA sequence coding, we need to consider the relationship between equilibrium unwinding temperature and stability, to ensure low unwinding temperature and high stability. As a rule of thumb, in experiments, the proportion of GC bases is usually set to 50%.

After analyzing the coding target and coding constraints, it is anticipated that lower the unwinding temperature and the similarity of the DNA strands will be, better, the constraint of GC bases will be limited to 50%, and the continuity will be lower to ensure the stability of DNA strands and easy synthesis. Hair should be avoided as much as possible. The evaluation criterion is defined as the equation (7).

$S=\left\{ \begin{aligned} S_{Tm}+S_{Sim}+S_{Hm}+S_{Con} GC=50\%Hair=0 \\ 0 GC\neq50\%Hair=0 \end{aligned} \right.$ (7)

Tm, Sim, Hm and Con are assigned according to the weights of 40,20,20 and 20 points. For DNA sequences with lower unwinding temperature, similarity, H-measure and continuity, the score is higher; for DNA sequences with GC content not equal to 50% or hairpin structure not equal to 0, the score is 0.

The vital idea of this approach is to create aptamers of ASC-speck by exploiting the heuristic principle of bacterial foraging optimization algorithm, so the method is likewise divided into four main stages, namely initialization, chemotaxis, replication and dispersal. With each DNA sequence mapped to each bacterium in the BFOA, after the above four main stages, some unfavorable DNA sequences can be eliminated under the condition that the total number remains unchanged, to obtain a better aptamer.


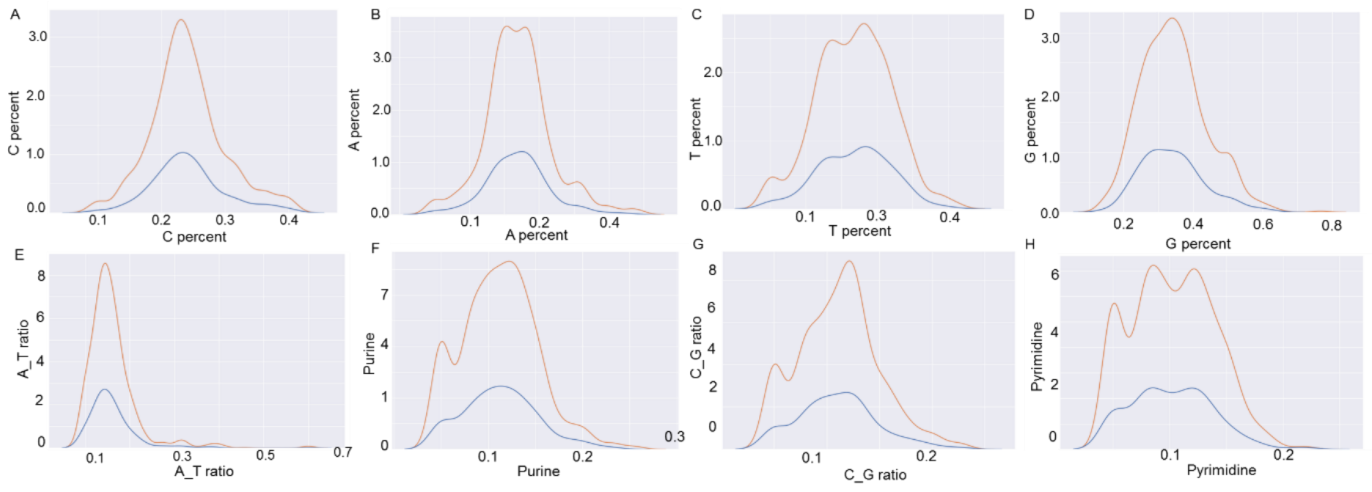


**Figure SI**. A-H. Plot of the DNA vs. aptamer features.


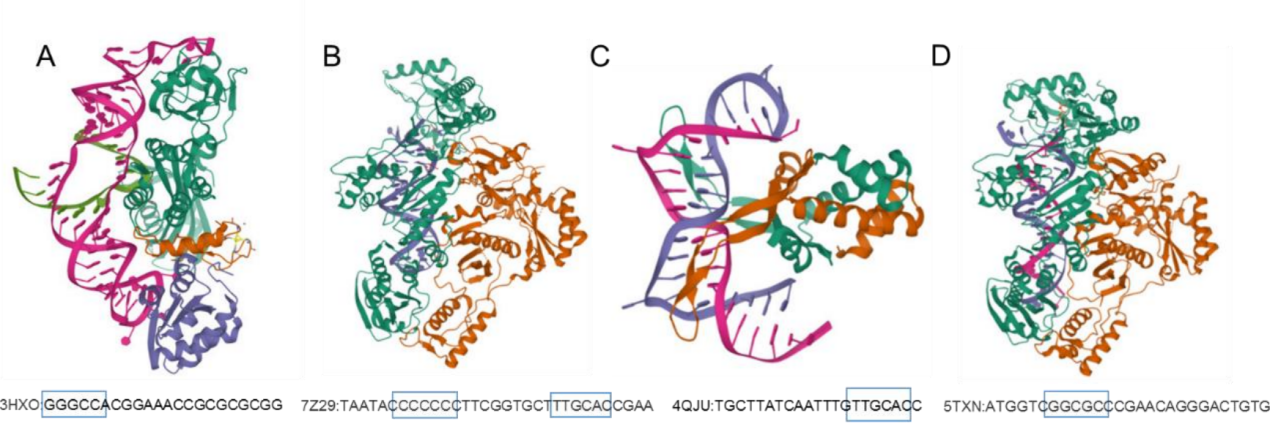


**Figure SII.** Structures of elucidated aptamers with their sequences. (A) 3HXO;(B) 7Z29;(C) 4QJU;(D)5TXN

**Table S1.** Comparison of existing aptamer predictive studies.

| Method | Aptamer dataset | Non-aptamer | Feature | Classifier | MCC |
| --- | --- | --- | --- | --- | --- |
| This  study | DNA aptamers :565 | Protein binding DNA 4885 | 6-mer, Pse-kNC, NMBAC for aptamers, physical-  chemical properties of targets | Deep multi- scale  ResNet | 0.915 |
| [1] | DNA aptamers 238 | Protein binding DNA 4885 | n-grams, sequence features | SVM | 0.896 |
| [2] | DNA/RNA | Randomly paired | 1,2-mer for aptamers | Nearest | 0.67 |
|  | aptamers 159 Small | aptamers to Small | physical-chemical | Neighbors |  |
|  | Molecule Targets 20 | Molecule targets | properties of targets |  |  |
| [3] | DNA/RNA | Randomly paired | 1,2-mer for targets physical- | Random | 0.461 |
|  | aptamers 725 | aptamers to Small | chemical properties of | Forest |  |
|  | Protein Target 164 | Molecule targets | targets |  |  |

**Table S2.** Comparison of existing aptamer predictive studies.

| Aptamer | Description |
| --- | --- |
| 3HXO[4] | The crystal structure of 3HXO is shown in Fig. SII(A). 3HXO is a 41-molecule DNA  aptamer that binds to the A1 domain of von Willebrand factor (VWF). The ligand has a  three-stem structure dominated by B-type DNA, consisting of three helical double rods and  two hairpin rings. It contains overlapping top predictions in the sequence 6-mers GGGCCA. |
| 7Z29 | The crystal structure of 7Z29 is shown in Fig. SII(B). 7Z29 is a 38-mer DNA aptamer. 7Z29  binds to HIV-1 reverse transcriptase (RT)[5]. It contains overlapping top predictions in the  sequence 6-mers CCCCCC and TTGCAC. |
| 4QJU | The crystal structure of 4QJU is shown in Fig. SII (C). 4QJU is a 21-molecule DNA  aptamer. 4QJU interacts with S. aureus SHU in a non-specific manner to induce DNA  bending or stabilize curved DNA. It contains overlapping top predictions in the  sequence 6-mers TTGCAC. |
| 5TXN | The crystal structure of 5TXN is shown in Fig. SII (D). 5TXN is a 41-molecule DNA  aptamer. It was associated with other exclusion mutations and enhanced RT's ability to  discriminate NRTIs other than dideoxyriboside[6]. It contains overlapping top predictions in  the sequence 6-mers GGCGCC. |

**References**

[1].Heredia, F.L., Roche-Lima, A. and Parés-Matos, E.I. (2021) A novel artificial intelligence-based approach for identification of deoxynucleotide aptamers, PLOS Computational Biology, 17, e1009247.

[2]Li, B.-Q., et al. (2014) Prediction of aptamer-target interacting pairs with pseudo-amino acid composition, PLoS One, 9, e86729.

[3]Muppirala, U.K., Honavar, V.G. and Dobbs, D. (2011) Predicting RNA-protein interactions using only sequence information, BMC bioinformatics, 12, 489.

[4]Huang R-H, Fremont DH, Diener JL, Schaub RG, Sadler JE (2009) A structural explanation for the antithrombotic activity of ARC1172, a DNA aptamer that binds von Willebrand factor domain A1. Structure 17: 1476-1484.

[5]. Singh AK, De Wijngaert B, Bijnens M, Uyttersprot K, Nguyen H, et al. (2022) Cryo-EM structures of wild- type and E138K/M184I mutant HIV-1 RT/DNA complexed with inhibitors doravirine and rilpivirine. Proceedings of the National Academy of Sciences 119: e2203660119.

[6]. Kim D-H, Im H, Jee J-G, Jang S-B, Yoon H-J, et al. (2014) β-Arm flexibility of HU from Staphylococcus aureus dictates the DNA-binding and recognition mechanism. Acta Crystallographica Section D: Biological Crystallography 70: 3273-3289.
